# Crystal structure and Hirshfeld surface analysis of 4,5-di­bromo-6-methyl-2-phenyl-2,3,3a,4,5,6,7,7a-octa­hydro-3a,6-ep­oxy-1*H*-isoindol-1-one

**DOI:** 10.1107/S205698902100116X

**Published:** 2021-02-09

**Authors:** Dmitriy F. Mertsalov, Maryana A. Nadirova, Lala V. Chervyakova, Mikhail S. Grigoriev, Evgeniya R. Shelukho, Sevim Türktekin Çelikesir, Mehmet Akkurt, Sixberth Mlowe

**Affiliations:** a Department of Organic Chemistry, Peoples’ Friendship University of Russia (RUDN University), 6 Miklukho-Maklaya St., 117198, Moscow, Russian Federation; b Frumkin Institute of Physical Chemistry and Electrochemistry, Russian Academy of Sciences, Leninsky pr. 31, bld. 4, Moscow, 119071, Russian Federation; c Department of Physics, Faculty of Sciences, Erciyes University, 38039 Kayseri, Turkey; d University of Dar es Salaam, Dar es Salaam University College of Education, Department of Chemistry, PO Box 2329, Dar es Salaam, Tanzania

**Keywords:** crystal structure, pyrrolidine ring, tetra­hydro­furan ring, ep­oxy­iso­indole moiety, Hirshfeld surface analysis

## Abstract

In the title compound, C_15_H_15_Br_2_NO_2_, two bridged tetra­hydro­furan rings adopt envelope conformations with the O atom as the flap. The pyrrolidine ring also adopts an envelope conformation with the spiro C atom as the flap. In the crystal, the mol­ecules are linked into dimers by pairs of C—H⋯O hydrogen bonds, thus generating *R* ^2^_2_(18) rings. The crystal packing is dominated by H⋯H, Br⋯H, H⋯π and Br⋯π inter­actions. One of the Br atoms is disordered over two sites with occupation ratio of 0.833 (8):0.167 (8).

## Abstract 2

In the crystal, mol­ecules are linked into dimers by pairs of C—H⋯O hydrogen bonds, thus generating 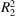
(18) rings. The crystal packing of the title compound is dominated by H⋯H, Br⋯H, H⋯π and Br⋯π inter­actions.

## Chemical context   

**Figure 1:**
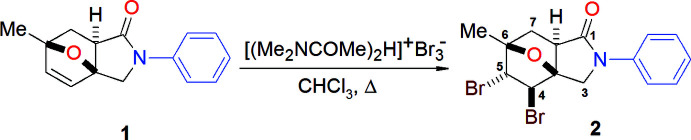
Synthesis scheme of 4,5-di­bromo-6-methyl-2-phenyl­hexa­hydro-3a,6-ep­oxy­isoindol-1(4*H*)-one (**2**).

The halogenation of oxabi­cyclo­heptenes plays an important role in the chemical transformations of bridged heterocycles because of the ability to carry out a complex transformation of the carbon skeleton in one step, which makes it possible to obtain products that are practically inaccessible in other ways from relatively simple starting compounds. The halogenation reaction of oxabi­cyclo­heptenes coupled with carbon- or nitro­gen-containing rings, with the help of various halogenating agents, proceeds in two possible general directions, depending on the nature of the halogenating agent and the structure of the substrate. Analysis of the literature data does not allow one to reliably predict the direction of the halogenation of oxabi­cyclo­heptenes. It can on the one hand be the halogen-initiated Wagner–Meerwein cationic rearrangement (Jung *et al.*, 1985 ▸; Ciganek *et al.*, 1995 ▸; Zubkov *et al.*, 2004 ▸, 2018 ▸; Zaytsev *et al.*, 2020 ▸), or on the other hand we can observe electrophilic addition of halogens to multiple bonds (Berson *et al.*, 1954 ▸; Barlow *et al.*, 1971 ▸; Kobayashi *et al.*, 1976 ▸; Solov’eva *et al.*, 1984 ▸). Halogenated organic compounds are of inter­est because of their photoactivity in the solid state, high solubility in halocarbons, high thermal and oxidative stability, *etc*., to which non-covalent halogen bonding can contribute (Afkhami *et al.*, 2017 ▸; Maharramov *et al.*, 2018 ▸; Mahmoudi *et al.*, 2017 ▸, 2019 ▸; Shixaliyev *et al.*, 2014 ▸). In view of its higher directionality, the halogen bond can be better suited than the hydrogen bond for the building of functional materials by non-covalent self-assembly *via* specific mol­ecular inter­actions (Gurbanov *et al.*, 2017 ▸, 2018 ▸; Kopylovich *et al.*, 2011 ▸; Ma *et al.*, 2017*a* ▸,*b* ▸, 2020 ▸; Mahmudov *et al.*, 2012 ▸, 2013 ▸, 2019 ▸, 2020 ▸). In a previous work (Zubkov *et al.*, 2018 ▸), the formation of a halogenated Wagner–Meervein rearrangement product under the action of mol­ecular bromine in dry di­chloro­methane on iso­indole **1** was shown. In this study, the effect of [(Me_2_NCOMe)_2_H]^+^Br_3_^−^ (Rodygin *et al.*, 1992 ▸; Prokop’eva *et al.*, 2008 ▸) is reported. The different course of the halogenation reaction was shown to be *anti*-addition on the double bond with the formation of the title compound, 4,5-di­bromo-6-methyl-2-phenyl­hexa­hydro-3a,6-ep­oxy-isoindol-1(4*H*)-one, **2** (Fig. 1 ▸).
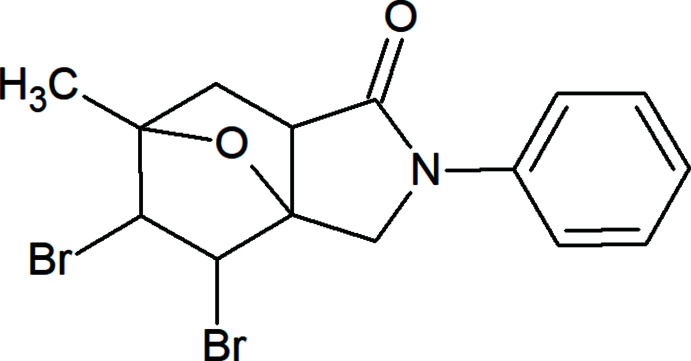


## Structural commentary   

**Figure 2:**
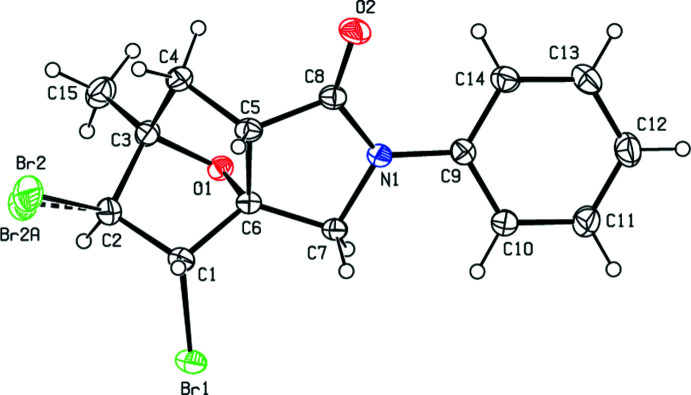
The mol­ecular structure of the title compound with displacement ellipsoids for the non-hydrogen atoms drawn at the 30% probability level. The atoms Br2 and Br2*A* represent the major and minor components of the disorder, respectively.

In the title compound (Fig. 2 ▸), the pyrrolidine ring (N1/C5–C8), tetra­hydro­furan rings (O1/C1–C3/C6 and O1/C3–C6) and the six-membered ring (C1–C6) that generate the ep­oxy­iso­indole moiety (O1/N1/C1–C8) are puckered (Cremer & Pople, 1975 ▸). Both tetra­hydro­furan rings adopt envelope conformations with puckering parameters of *Q*(2) = 0.5749 (14) Å, φ(2) = 0.92 (16)° for (O1/C1–C3/C6) and *Q*(2) = 0.5460 (14) Å, φ(2) = 183.90 (17)° for (O1/C3–C6). The five-membered pyrrolidine ring has an envelope conformation with a maximum deviation from the mean plane of 0.166 (1) Å at C6 [puckering parameters *Q*(2) = 0.2630 (16) Å, φ(2) = 253.9 (3)°]. The six-membered ring (C1–C6) has a boat conformation [*Q*_T_ = 0.9320 (16) Å, θ = 88.92 (10)°, φ = 298.57 (10)°]. The Br2 atom is disordered over two sites with occupation ratio of 0.833 (8):0.167 (8).

## Supra­molecular features   

**Figure 3:**
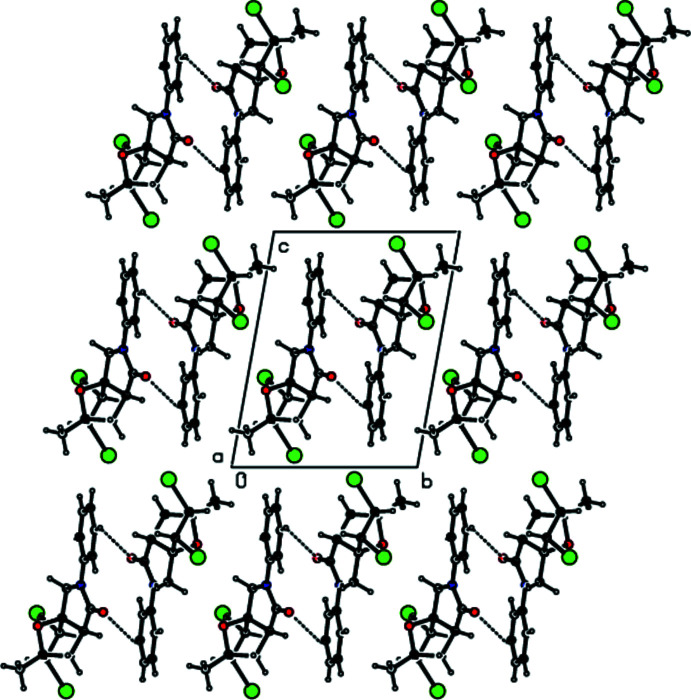
A view of the inter­molecular C—H⋯O inter­actions in the crystal structure of the title compound. Only the major component of the disorder is shown.

**Figure 4:**
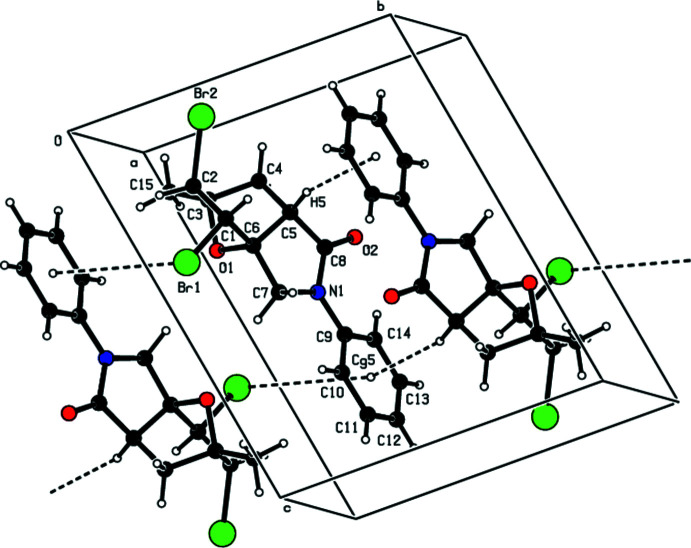
A view of the inter­molecular C—H⋯π and C—Br⋯π inter­actions in the crystal structure of the title compound. Only the major component of the disorder is shown.

The crystal packing of the title compound is consolidated by C—H⋯O hydrogen bonds (Table 1 ▸, Fig. 3 ▸) and C—H⋯π and C—Br⋯π inter­actions (Table 1 ▸, Fig. 4 ▸). In the crystal, pairs of C—H⋯O hydrogen bonds link mol­ecules into dimers with 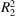
(18) ring motifs (Bernstein *et al.* 1995 ▸). These dimers are connected by pairs of C—H⋯π inter­actions and C—Br⋯π inter­actions [Br1⋯*Cg*5^iii^ = 3.9246 (8) Å, C1—Br1⋯*Cg*5^iii^ = 112.92 (5)°; symmetry code: (iii) 1 − *x*, −*y*, 1 − *z*], thus forming layers parallel to the *ab* plane. Short atomic contacts are listed in Table 2 ▸.

## Hirshfeld surface analysis   

**Figure 5:**
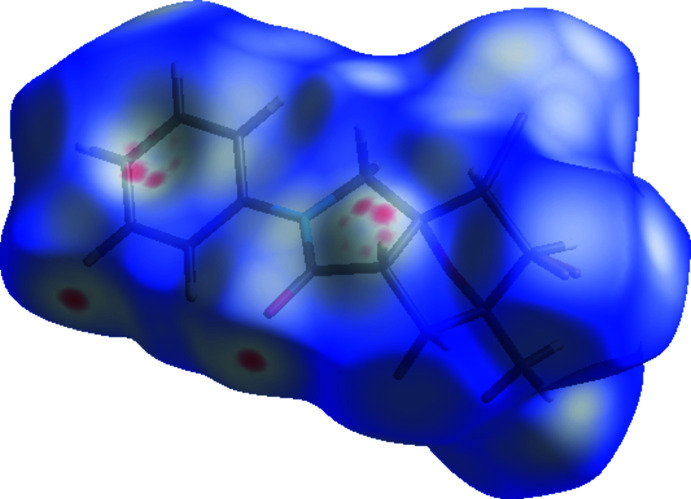
A view of the three-dimensional Hirshfeld surface for the title compound, plotted over *d*_norm_ in the range −0.1151 to 1.1998 a.u.

**Figure 6:**
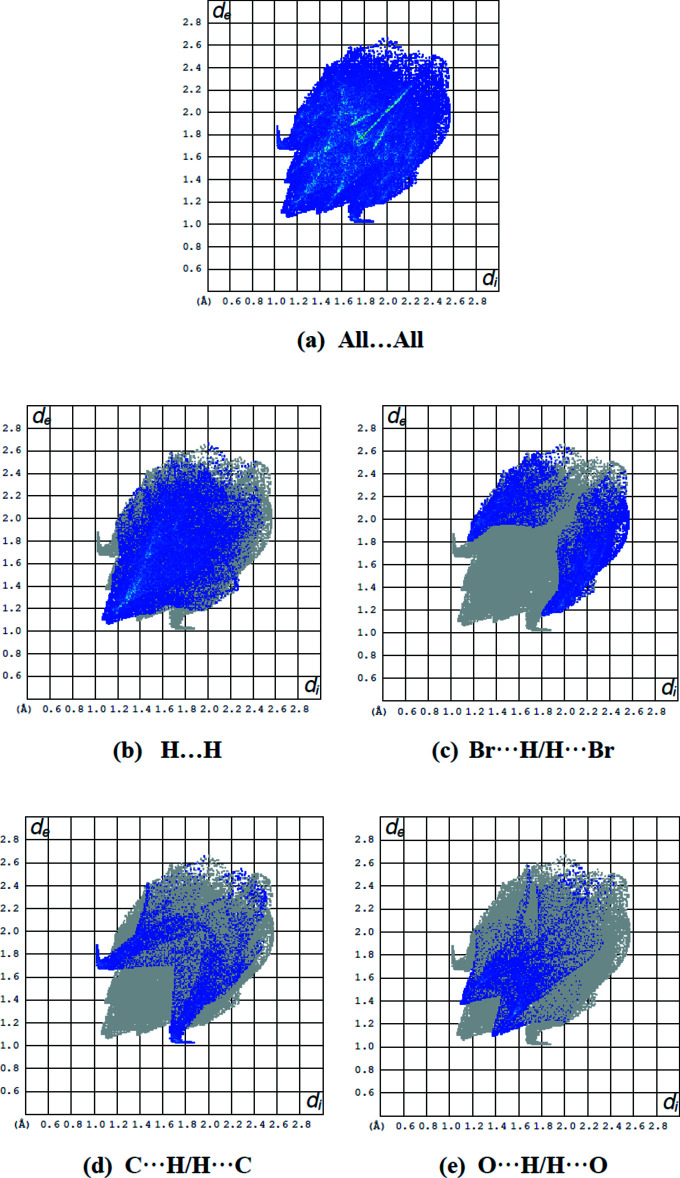
A view of the two-dimensional fingerprint plots for the title compound, showing (*a*) all inter­actions, and delineated into (*b*) H⋯H, (*c*) Br⋯H/H⋯Br, (*d*) C⋯H/H⋯C and (*e*) O⋯H/H⋯O inter­actions. The *d*_i_ and *d*_e_ values are the closest inter­nal and external distances (in Å) from given points on the Hirshfeld surface.

In order to present the inter­molecular inter­actions in the crystal structure of the title compound in a visual manner, Hirshfeld surfaces (McKinnon *et al.*, 2007 ▸) and their associated two-dimensional fingerprint plots (Spackman & McKinnon, 2002 ▸) were generated using *CrystalExplorer17* (Turner *et al.*, 2017 ▸). The Hirshfeld surface plotted over *d*_norm_ in the range −0.1151 to 1.1998 a.u. is shown in Fig. 5 ▸ while Fig. 6 ▸ shows the full two-dimensional fingerprint plot and those delineated into the major contacts: H⋯H (43.0%), Br⋯H/H⋯Br (21.1%), C⋯H/H⋯C (12.4%) and O⋯H/H⋯O (11.9%). The other contacts (Table 3 ▸) are negligible with individual contributions of less than 3.5% and a sum of less than 11.5%.

## Database survey   

A search of the Cambridge Crystallographic Database (CSD version 5.40, update of September 2019; Groom *et al.*, 2016 ▸) yielded six entries closely related to the ep­oxy­iso­indole moiety of the title compound, *viz*.: (3a*R*,6*S*,7a*R*)-7a-bromo-2-methyl­sulfonyl-1,2,3,6,7,7a-hexa­hydro-3a,6-ep­oxy­iso­indole (CSD refcode ERIVIL; Temel *et al.*, 2011 ▸), (3a*R*,6*S*,7a*R*)-7a-chloro-2-[(4-nitro­phen­yl)sulfon­yl]-1,2,3,6,7,7a-hexa­hydro-3a,6-ep­oxy­iso­indole (AGONUH; Temel *et al.*, 2013 ▸), (3a*R*,6*S*,7a*R*)-7a-chloro-6-methyl-2-[(4-nitro­phen­yl)sulfon­yl]-1,2,3,6,7,7a-hexa­hydro-3a,6-ep­oxy­iso­indole (TIJMIK; Demir­can *et al.*, 2013 ▸), (3a*R*,6*S*,7a*R*)-7a-bromo-2-[(4-methylphen­yl)sulfon­yl]-1,2,3,6,7,7a-hexa­hydro-3a,6-ep­oxy­iso­indole (UPAQEI; Koşar *et al.*, 2011 ▸), 5-chloro-7-methyl-3-[(4-methyl­phen­yl)sulfon­yl]-10-oxa-3-aza­tri­cyclo­[5.2.1.01,5]dec-8-ene (YAXCIL; Temel *et al.*, 2012 ▸) and *tert*-butyl 3a-chloro­perhydro-2,6a-ep­oxy­oxireno(*e*)iso­indole-5-carboxyl­ate (MIG­TIG; Koşar *et al.*, 2007 ▸).

In the crystal of ERIVIL, weak inter­molecular C—H⋯O hydrogen bonds link the mol­ecules into 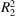
(8) and 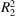
(14) rings, thus forming the chains along the *b*-axis direction. In the crystal of AGONUH, C—H⋯O hydrogen bonds link the mol­ecules into zigzag chains running along the *b*-axis direction. In TIJMIK, two types of C—H⋯O hydrogen bonds generate 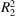
(20) and 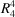
(26) rings, with adjacent rings running parallel to the *ac* plane. Further C—H⋯O hydrogen bonds form a *C*(6) chain, linking the mol­ecules in the *b*-axis direction. In UPAQEI, mol­ecules are linked by C—H⋯O hydrogen bonds. In YAXCIL, C—H⋯O hydrogen bonds link the mol­ecules into a three-dimensional network. In MIGTIG, the mol­ecules are linked only by weak van der Waals inter­actions.

## Synthesis and crystallization   

The solution of isoindolone **1** (4 mmol) and the brominating agent (4 mmol) in 15 mL of dry chloro­form was heated under reflux for 20 h (TLC control, EtOAc–hexane, 1:1). The reaction mixture was poured into H_2_O (50 mL) and extracted with CHCl_3_ (3 × 20 mL). The combined organic fractions were dried over anhydrous Na_2_SO_4_, the solvent was evaporated under reduced pressure, and the solid residue was recrystallized from a hexa­ne–AcOEt (1:1) mixture in the form of colourless needles [yield 0.48 g (30%), m.p. > 413 K (decomposition)].

IR (KBr), ν (cm^−1^): 1700 (N—C=O), 689 (C—Br). ^1^H NMR (CDCl_3_, 600.2 MHz, 301 K): δ = 7.63 (*d*, 2H, H2, H6, HAr, *J =* 7.6), 7.39 (*t*, 2H, H3, H5, HAr, *J =* 7.6), 7.19 (*t*, 1H, H4, HAr, *J* = 7.6), 4.33 (*d*, 1H, H4, *J* = 2.2), 4.24 (*t*, 1H, H5, *J* = 2.2), 4.07 (*d*, 1H, *J* = 11.8), 4.02 (*d*, 1H, H3, *J =* 11.8), 3.00 (*dd*, 1H, H7a, *J =* 5.0, *J =* 9.6), 2.85 (*dd*, 1H, H7*B*, *J =* 9.6, *J =* 13.1), 2.07 (*ddd*, 1H, H7*A*, *J =* 2.2, *J =* 5.0, *J =* 13.1), 1.58 (*s*, 3H, CH_3_). ^13^C NMR (CDCl_3_, 150.9 MHz, 301 K): δ = 172.4, 138.7, 129.0 (2C), 125.1, 120.1 (2C), 89.5, 88.0, 60.4, 57.0, 51.1, 51.1, 36.0, 18.1. MS (APCI): *m*/*z* = 404 [*M* + H]^+^ (^81^Br), 402 [*M* + H]^+^ (^81^Br, ^79^Br), 400 [*M* + H]^+^ (^79^Br).

## Refinement   

Crystal data, data collection and structure refinement details are summarized in Table 4 ▸. All the C-bound H atoms were positioned geometrically, with C—H = 0.93 Å (for aromatic H atoms), 0.98 Å (for methine H atoms), 0.97 Å (for methyl­ene H atoms) and 0.96 Å (for methyl H atoms), and constrained to ride on their parent atoms, with *U*_iso_(H) = 1.2*U*_eq_(C) [1.5*U*_eq_(C) for methyl H atoms]. The Br2 atom attached to the atom C2 is disordered over two sites, with occupancies of 0.833 (8)/0.167 (8). The two components of the disorder (Br2 and Br2*A*) were refined with restraints so that their bond lengths are comparable. Owing to poor agreement, five reflections, *i.e*. (126), (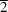
04), (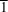 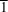
5), (321) and (006), were omitted from the final cycles of refinement.

## Supplementary Material

Crystal structure: contains datablock(s) I. DOI: 10.1107/S205698902100116X/yk2144sup1.cif

Structure factors: contains datablock(s) I. DOI: 10.1107/S205698902100116X/yk2144Isup2.hkl

Supporting information file. DOI: 10.1107/S205698902100116X/yk2144Isup3.cml

CCDC reference: 2060298

Additional supporting information:  crystallographic information; 3D view; checkCIF report

## Figures and Tables

**Table 1:** Hydrogen-bond geometry (Å, °) *Cg*5 is the centroid of the C9–C14 ring.

*D*—H⋯*A*	*D*—H	H⋯*A*	*D*⋯*A*	*D*—H⋯*A*
C13—H13⋯O2^i^	0.93	2.58	3.223 (2)	127
C5—H5⋯*Cg*5^ii^	0.98	2.49	3.4195 (17)	158

Symmetry codes: (i) -x+2, -y+1, -z+1; (ii) -x+1, -y+1, -z+1.

**Table 2:** Summary of short inter­atomic contacts (Å) in the title compound

Contact	Distance	Symmetry operation
H7*A*⋯H14	2.56	−1 + *x*, *y*, *z*
Br1⋯Br1	3.4852 (3)	−*x*, −*y*, 1 − *z*
H15*C*⋯H10	2.53	1 − *x*, −*y*, 1 − *z*
H15*B*⋯H11	2.40	*x*, *y*, −1 + *z*
Br2*A*⋯H12	3.13	−1 + *x*, *y*, −1 + *z*
H5⋯C14	2.83	1 − *x*, 1 − *y*, 1 − *z*
H13⋯O2	2.58	2 − *x*, 1 − *y*, 1 − *z*

**Table 3:** Percentage contributions of inter­atomic contacts to the Hirshfeld surface for the title compound

Contact	Percentage contribution
H⋯H	43.0
Br⋯H/H⋯Br	21.1
C⋯H/H⋯C	12.4
O⋯H/H⋯O	11.9
Br⋯C/C⋯Br	3.5
Br⋯Br	2.9
Br⋯O/O⋯Br	2.5
Br⋯N/N⋯Br	1.1
C⋯C	0.5
C⋯N/N⋯C	0.5
C⋯O/O⋯C	0.3
N⋯O/O⋯N	0.1
N⋯N	0.1

**Table 4:** Experimental details

Crystal data	
Chemical formula	C_15_H_15_Br_2_NO_2_
*M*_r_	401.10
Crystal system, space group	Triclinic, *P*\overline{1}
Temperature (K)	296
*a*, *b*, *c* (Å)	6.8064 (2), 9.5045 (2), 11.9482 (3)
α, β, γ (°)	79.551 (1), 87.820 (1), 77.083 (1)
*V* (Å^3^)	740.89 (3)
*Z*	2
Radiation type	Mo *K*α
μ (mm^−1^)	5.47
Crystal size (mm)	0.14 × 0.13 × 0.13

Data collection	
Diffractometer	Bruker Kappa APEXII area-detector diffractometer
Absorption correction	Multi-scan (*SADABS*; Krause *et al.*, 2015 ▸)
*T*_min_, *T*_max_	0.184, 0.273
No. of measured, independent and observed [*I* > 2σ(*I*)] reflections	19111, 4387, 3575
*R*_int_	0.025
(sin θ/λ)_max_ (Å^−1^)	0.711

Refinement	
*R*[*F* ^2^ > 2σ(*F* ^2^)], *wR*(*F* ^2^), *S*	0.025, 0.059, 1.05
No. of reflections	4387
No. of parameters	187
No. of restraints	2
H-atom treatment	H-atom parameters constrained
Δρ_max_, Δρ_min_ (e Å^−3^)	0.34, −0.36

Computer programs: *APEX2* and *SAINT* (Bruker, 2013 ▸), *SHELXT* (Sheldrick, 2015*a* ▸), *SHELXL* (Sheldrick, 2015*b* ▸), *ORTEP-3 for Windows* (Farrugia, 2012 ▸) and *PLATON* (Spek, 2020 ▸).

## supplementary crystallographic information

### Crystal data

**Table:**

C_15_H_15_Br_2_NO_2_	*Z* = 2
*M**_r_* = 401.10	*F*(000) = 396
Triclinic, *P*1	*D*_x_ = 1.798 Mg m^−^^3^
*a* = 6.8064 (2) Å	Mo *K*α radiation, λ = 0.71073 Å
*b* = 9.5045 (2) Å	Cell parameters from 9338 reflections
*c* = 11.9482 (3) Å	θ = 2.6–29.2°
α = 79.551 (1)°	µ = 5.47 mm^−^^1^
β = 87.820 (1)°	*T* = 296 K
γ = 77.083 (1)°	Fragment, colourless
*V* = 740.89 (3) Å^3^	0.14 × 0.13 × 0.13 mm

### Data collection

**Table:**

Bruker Kappa APEXII area-detector diffractometer	3575 reflections with *I* > 2σ(*I*)
ω– and φ–scans	*R*_int_ = 0.025
Absorption correction: multi-scan (SADABS; Krause *et al.*, 2015)	θ_max_ = 30.3°, θ_min_ = 3.8°
*T*_min_ = 0.184, *T*_max_ = 0.273	*h* = −8→9
19111 measured reflections	*k* = −13→13
4387 independent reflections	*l* = −16→16

### Refinement

**Table:**

Refinement on *F*^2^	Hydrogen site location: inferred from neighbouring sites
Least-squares matrix: full	H-atom parameters constrained
*R*[*F*^2^ > 2σ(*F*^2^)] = 0.025	*w* = 1/[σ^2^(*F*_o_^2^) + (0.0265*P*)^2^ + 0.1676*P*] where *P* = (*F*_o_^2^ + 2*F*_c_^2^)/3
*wR*(*F*^2^) = 0.059	(Δ/σ)_max_ = 0.001
*S* = 1.05	Δρ_max_ = 0.34 e Å^−^^3^
4387 reflections	Δρ_min_ = −0.35 e Å^−^^3^
187 parameters	Extinction correction: SHELXL-2018/3 (Sheldrick 2008), Fc^*^=kFc[1+0.001xFc^2^λ^3^/sin(2θ)]^-1/4^
2 restraints	Extinction coefficient: 0.0099 (8)

### Special details

**Table:**

Geometry. All esds (except the esd in the dihedral angle between two l.s. planes) are estimated using the full covariance matrix. The cell esds are taken into account individually in the estimation of esds in distances, angles and torsion angles; correlations between esds in cell parameters are only used when they are defined by crystal symmetry. An approximate (isotropic) treatment of cell esds is used for estimating esds involving l.s. planes.

### Fractional atomic coordinates and isotropic or equivalent isotropic displacement parameters (Å^2^)

**Table:**

	*x*	*y*	*z*	*U*_iso_*/*U*_eq_	Occ. (<1)
C1	0.2491 (2)	0.23465 (16)	0.29816 (13)	0.0300 (3)	
H1	0.162569	0.333375	0.282028	0.036*	
C2	0.3412 (2)	0.18494 (19)	0.18835 (14)	0.0358 (3)	
H2	0.312544	0.089368	0.184511	0.043*	
C3	0.5718 (2)	0.16536 (17)	0.20591 (13)	0.0315 (3)	
C4	0.6318 (2)	0.31286 (17)	0.19882 (13)	0.0346 (3)	
H4A	0.580003	0.380680	0.130185	0.041*	
H4B	0.776935	0.300779	0.202053	0.041*	
C5	0.5290 (2)	0.36343 (16)	0.30594 (13)	0.0302 (3)	
H5	0.423305	0.452582	0.285765	0.036*	
C6	0.4389 (2)	0.23380 (15)	0.36065 (12)	0.0264 (3)	
C7	0.4433 (2)	0.23050 (17)	0.48669 (13)	0.0289 (3)	
H7A	0.316869	0.284391	0.512777	0.035*	
H7B	0.470492	0.130584	0.528351	0.035*	
C8	0.6637 (2)	0.37833 (16)	0.39896 (13)	0.0303 (3)	
C9	0.6869 (2)	0.29591 (16)	0.60907 (13)	0.0292 (3)	
C10	0.5782 (3)	0.24987 (18)	0.70424 (14)	0.0356 (3)	
H10	0.456428	0.224227	0.695094	0.043*	
C11	0.6499 (3)	0.2420 (2)	0.81232 (15)	0.0449 (4)	
H11	0.575891	0.211181	0.875398	0.054*	
C12	0.8306 (3)	0.2794 (2)	0.82762 (17)	0.0480 (4)	
H12	0.878984	0.273335	0.900469	0.058*	
C13	0.9379 (3)	0.3257 (2)	0.73344 (17)	0.0451 (4)	
H13	1.059246	0.351480	0.743341	0.054*	
C14	0.8691 (2)	0.33463 (18)	0.62468 (15)	0.0374 (4)	
H14	0.943622	0.366258	0.562061	0.045*	
C15	0.7046 (3)	0.0567 (2)	0.14292 (16)	0.0463 (4)	
H15A	0.841396	0.039666	0.168395	0.069*	
H15B	0.697046	0.095089	0.062760	0.069*	
H15C	0.660246	−0.033910	0.157404	0.069*	
N1	0.60928 (18)	0.30218 (13)	0.49983 (11)	0.0289 (3)	
O1	0.57998 (14)	0.11085 (10)	0.32711 (8)	0.0280 (2)	
O2	0.79438 (19)	0.44882 (13)	0.38590 (11)	0.0435 (3)	
Br1	0.10285 (2)	0.09342 (2)	0.38126 (2)	0.03963 (7)	
Br2	0.22893 (10)	0.3267 (3)	0.05089 (6)	0.0579 (2)	0.833 (8)
Br2A	0.2336 (6)	0.2821 (8)	0.0560 (3)	0.0579 (2)	0.167 (8)

### Atomic displacement parameters (Å^2^)

**Table:**

	*U*^11^	*U*^22^	*U*^33^	*U*^12^	*U*^13^	*U*^23^
C1	0.0257 (7)	0.0304 (7)	0.0335 (8)	−0.0070 (6)	−0.0009 (6)	−0.0037 (6)
C2	0.0354 (8)	0.0430 (9)	0.0299 (8)	−0.0118 (7)	−0.0030 (6)	−0.0043 (7)
C3	0.0308 (7)	0.0367 (8)	0.0263 (7)	−0.0063 (6)	0.0015 (6)	−0.0053 (6)
C4	0.0350 (8)	0.0404 (8)	0.0284 (8)	−0.0137 (7)	0.0021 (6)	0.0000 (6)
C5	0.0295 (7)	0.0273 (7)	0.0326 (8)	−0.0081 (6)	0.0006 (6)	−0.0002 (6)
C6	0.0237 (6)	0.0262 (7)	0.0291 (7)	−0.0060 (5)	0.0020 (5)	−0.0044 (5)
C7	0.0261 (7)	0.0327 (7)	0.0302 (7)	−0.0114 (6)	0.0041 (6)	−0.0067 (6)
C8	0.0300 (7)	0.0270 (7)	0.0344 (8)	−0.0086 (6)	0.0022 (6)	−0.0048 (6)
C9	0.0292 (7)	0.0265 (7)	0.0322 (8)	−0.0048 (6)	−0.0010 (6)	−0.0074 (6)
C10	0.0376 (8)	0.0386 (8)	0.0333 (8)	−0.0148 (7)	0.0013 (7)	−0.0060 (7)
C11	0.0539 (11)	0.0509 (10)	0.0320 (9)	−0.0194 (8)	−0.0006 (8)	−0.0031 (7)
C12	0.0547 (11)	0.0540 (11)	0.0369 (9)	−0.0164 (9)	−0.0127 (8)	−0.0041 (8)
C13	0.0349 (9)	0.0517 (10)	0.0505 (11)	−0.0124 (8)	−0.0104 (8)	−0.0083 (8)
C14	0.0295 (8)	0.0424 (9)	0.0417 (9)	−0.0092 (7)	0.0012 (7)	−0.0096 (7)
C15	0.0462 (10)	0.0526 (10)	0.0394 (10)	−0.0059 (8)	0.0088 (8)	−0.0145 (8)
N1	0.0282 (6)	0.0313 (6)	0.0298 (6)	−0.0116 (5)	0.0019 (5)	−0.0068 (5)
O1	0.0269 (5)	0.0275 (5)	0.0280 (5)	−0.0034 (4)	0.0020 (4)	−0.0040 (4)
O2	0.0445 (7)	0.0450 (7)	0.0462 (7)	−0.0265 (5)	−0.0004 (5)	−0.0003 (5)
Br1	0.03119 (9)	0.04352 (10)	0.04675 (11)	−0.01621 (7)	0.00310 (7)	−0.00536 (7)
Br2	0.05211 (13)	0.0780 (7)	0.03458 (13)	−0.0074 (3)	−0.01400 (9)	0.0075 (2)
Br2A	0.05211 (13)	0.0780 (7)	0.03458 (13)	−0.0074 (3)	−0.01400 (9)	0.0075 (2)

### Geometric parameters (Å, º)

**Table:**

C1—C6	1.514 (2)	C7—H7A	0.9700
C1—C2	1.539 (2)	C7—H7B	0.9700
C1—Br1	1.9538 (15)	C8—O2	1.2175 (18)
C1—H1	0.9800	C8—N1	1.3722 (19)
C2—C3	1.556 (2)	C9—C10	1.390 (2)
C2—Br2A	1.773 (4)	C9—C14	1.398 (2)
C2—Br2	1.982 (2)	C9—N1	1.413 (2)
C2—H2	0.9800	C10—C11	1.381 (2)
C3—O1	1.4455 (18)	C10—H10	0.9300
C3—C15	1.504 (2)	C11—C12	1.381 (3)
C3—C4	1.533 (2)	C11—H11	0.9300
C4—C5	1.537 (2)	C12—C13	1.377 (3)
C4—H4A	0.9700	C12—H12	0.9300
C4—H4B	0.9700	C13—C14	1.380 (3)
C5—C8	1.513 (2)	C13—H13	0.9300
C5—C6	1.529 (2)	C14—H14	0.9300
C5—H5	0.9800	C15—H15A	0.9600
C6—O1	1.4445 (16)	C15—H15B	0.9600
C6—C7	1.502 (2)	C15—H15C	0.9600
C7—N1	1.4699 (19)		
			
C6—C1—C2	100.18 (11)	N1—C7—C6	102.98 (11)
C6—C1—Br1	111.46 (10)	N1—C7—H7A	111.2
C2—C1—Br1	110.81 (10)	C6—C7—H7A	111.2
C6—C1—H1	111.3	N1—C7—H7B	111.2
C2—C1—H1	111.3	C6—C7—H7B	111.2
Br1—C1—H1	111.3	H7A—C7—H7B	109.1
C1—C2—C3	103.47 (12)	O2—C8—N1	126.42 (15)
C1—C2—Br2A	118.5 (2)	O2—C8—C5	125.35 (14)
C3—C2—Br2A	118.35 (18)	N1—C8—C5	108.22 (12)
C1—C2—Br2	111.70 (12)	C10—C9—C14	118.90 (15)
C3—C2—Br2	114.67 (11)	C10—C9—N1	118.84 (14)
C1—C2—H2	108.9	C14—C9—N1	122.25 (14)
C3—C2—H2	108.9	C11—C10—C9	120.38 (16)
Br2—C2—H2	108.9	C11—C10—H10	119.8
O1—C3—C15	110.86 (13)	C9—C10—H10	119.8
O1—C3—C4	101.92 (12)	C12—C11—C10	120.66 (17)
C15—C3—C4	116.12 (14)	C12—C11—H11	119.7
O1—C3—C2	98.23 (11)	C10—C11—H11	119.7
C15—C3—C2	115.34 (14)	C13—C12—C11	119.02 (18)
C4—C3—C2	111.95 (13)	C13—C12—H12	120.5
C3—C4—C5	100.86 (12)	C11—C12—H12	120.5
C3—C4—H4A	111.6	C12—C13—C14	121.36 (17)
C5—C4—H4A	111.6	C12—C13—H13	119.3
C3—C4—H4B	111.6	C14—C13—H13	119.3
C5—C4—H4B	111.6	C13—C14—C9	119.67 (16)
H4A—C4—H4B	109.4	C13—C14—H14	120.2
C8—C5—C6	102.91 (12)	C9—C14—H14	120.2
C8—C5—C4	117.40 (13)	C3—C15—H15A	109.5
C6—C5—C4	102.89 (12)	C3—C15—H15B	109.5
C8—C5—H5	111.0	H15A—C15—H15B	109.5
C6—C5—H5	111.0	C3—C15—H15C	109.5
C4—C5—H5	111.0	H15A—C15—H15C	109.5
O1—C6—C7	112.07 (12)	H15B—C15—H15C	109.5
O1—C6—C1	102.18 (11)	C8—N1—C9	126.80 (13)
C7—C6—C1	122.52 (12)	C8—N1—C7	112.86 (12)
O1—C6—C5	102.27 (11)	C9—N1—C7	120.22 (12)
C7—C6—C5	105.82 (12)	C6—O1—C3	97.27 (10)
C1—C6—C5	110.27 (12)		
			
C6—C1—C2—C3	−0.64 (15)	O1—C6—C7—N1	85.18 (13)
Br1—C1—C2—C3	−118.41 (11)	C1—C6—C7—N1	−152.93 (13)
C6—C1—C2—Br2A	−133.9 (3)	C5—C6—C7—N1	−25.51 (14)
Br1—C1—C2—Br2A	108.3 (3)	C6—C5—C8—O2	164.98 (15)
C6—C1—C2—Br2	−124.51 (12)	C4—C5—C8—O2	52.9 (2)
Br1—C1—C2—Br2	117.72 (11)	C6—C5—C8—N1	−16.51 (15)
C1—C2—C3—O1	35.54 (14)	C4—C5—C8—N1	−128.64 (14)
Br2A—C2—C3—O1	168.9 (3)	C14—C9—C10—C11	−0.3 (2)
Br2—C2—C3—O1	157.45 (12)	N1—C9—C10—C11	179.79 (15)
C1—C2—C3—C15	153.36 (14)	C9—C10—C11—C12	−0.1 (3)
Br2A—C2—C3—C15	−73.3 (3)	C10—C11—C12—C13	0.4 (3)
Br2—C2—C3—C15	−84.74 (17)	C11—C12—C13—C14	−0.3 (3)
C1—C2—C3—C4	−70.91 (15)	C12—C13—C14—C9	−0.1 (3)
Br2A—C2—C3—C4	62.4 (3)	C10—C9—C14—C13	0.4 (2)
Br2—C2—C3—C4	50.99 (17)	N1—C9—C14—C13	−179.71 (15)
O1—C3—C4—C5	−36.84 (14)	O2—C8—N1—C9	3.2 (3)
C15—C3—C4—C5	−157.42 (14)	C5—C8—N1—C9	−175.27 (13)
C2—C3—C4—C5	67.22 (15)	O2—C8—N1—C7	179.05 (15)
C3—C4—C5—C8	115.57 (14)	C5—C8—N1—C7	0.57 (17)
C3—C4—C5—C6	3.44 (14)	C10—C9—N1—C8	161.81 (15)
C2—C1—C6—O1	−34.97 (13)	C14—C9—N1—C8	−18.1 (2)
Br1—C1—C6—O1	82.31 (11)	C10—C9—N1—C7	−13.8 (2)
C2—C1—C6—C7	−161.36 (13)	C14—C9—N1—C7	166.34 (14)
Br1—C1—C6—C7	−44.08 (16)	C6—C7—N1—C8	15.92 (16)
C2—C1—C6—C5	73.18 (14)	C6—C7—N1—C9	−167.93 (12)
Br1—C1—C6—C5	−169.54 (10)	C7—C6—O1—C3	−167.25 (12)
C8—C5—C6—O1	−91.53 (13)	C1—C6—O1—C3	59.84 (12)
C4—C5—C6—O1	30.94 (14)	C5—C6—O1—C3	−54.35 (13)
C8—C5—C6—C7	25.94 (15)	C15—C3—O1—C6	−178.76 (13)
C4—C5—C6—C7	148.41 (12)	C4—C3—O1—C6	57.05 (12)
C8—C5—C6—C1	160.39 (12)	C2—C3—O1—C6	−57.57 (12)
C4—C5—C6—C1	−77.14 (14)		

### Hydrogen-bond geometry (Å, º)

*Cg*5 is the centroid of the C9–C14 ring.

**Table:**

*D*—H···*A*	*D*—H	H···*A*	*D*···*A*	*D*—H···*A*
C4—H4*A*···Br2	0.97	2.79	3.2838 (17)	113
C13—H13···O2^i^	0.93	2.58	3.223 (2)	127
C14—H14···O2	0.93	2.30	2.884 (2)	120
C5—H5···*Cg*5^ii^	0.98	2.49	3.4195 (17)	158

Symmetry codes: (i) −*x*+2, −*y*+1, −*z*+1; (ii) −*x*+1, −*y*+1, −*z*+1.
